# Secondary pulmonary alveolar proteinosis mimicking progression of interstitial lung disease during treatment for adult-onset still’s disease: An autopsy case report

**DOI:** 10.1097/MD.0000000000051085

**Published:** 2026-10-09

**Authors:** Takashi Yamane, Taisuke Fujita, Atsushi Onoe, Chihiro Ichikawa

**Affiliations:** a Department of Rheumatology, Kakogawa Central City Hospital, Kakogawa, Hyogo, Japan; b Department of Surgical Pathology, Kakogawa Central City Hospital, Kakogawa, Hyogo, Japan.

**Keywords:** Adult-onset Still’s disease, Connective tissue disease–associated interstitial lung disease, Immunosuppressive therapy, KL-6, Secondary pulmonary alveolar proteinosis

## Abstract

**Rationale:**

Secondary pulmonary alveolar proteinosis (sPAP) is a rare disorder caused by impaired surfactant clearance due to alveolar macrophage dysfunction and is often associated with hematologic disorders or immunosuppressive therapy. In patients with connective tissue disease–associated interstitial lung disease (CTD-ILD), worsening respiratory findings are frequently interpreted as progression of the underlying ILD, which may delay recognition of sPAP, particularly when anti–granulocyte-macrophage colony-stimulating factor (GM-CSF) antibodies are negative. In the present case, preexisting ILD and repeated bronchoscopic findings of organizing pneumonia further obscured the development of sPAP, making the diagnosis particularly challenging.

**Patient concerns:**

A 68-year-old woman with adult-onset Still’s disease (AOSD)–associated ILD developed progressive respiratory deterioration during immunosuppressive therapy. Despite apparent control of AOSD-related systemic inflammation, serum Krebs von den Lungen-6 (KL-6) levels increased markedly, and new diffuse ground-glass opacities appeared on chest computed tomography.

**Diagnoses:**

Repeated bronchoscopic examinations demonstrated organizing pneumonia without features suggestive of PAP, supporting the interpretation of progressive CTD-ILD. However, KL-6 levels progressively increased to 19,333 IU per liter despite treatment, raising suspicion of PAP. Anti–GM-CSF antibody testing was negative, and a definitive diagnosis could not be established during life. Autopsy ultimately revealed sPAP with periodic acid–Schiff–positive intra-alveolar material and myelodysplastic syndrome (MDS). Hematologic abnormalities had initially been attributed to systemic inflammation, and subsequent bone marrow examination showed MDS-like dysplasia without establishing a definitive diagnosis of MDS.

**Interventions:**

The patient received escalating immunosuppressive therapies, including a Janus kinase inhibitor, under the assumption of progressive CTD-ILD, with the aim of minimizing glucocorticoid exposure.

**Outcomes:**

Respiratory failure progressed despite treatment, and bronchoscopy was not feasible in the terminal stage. The patient died of respiratory failure. Postmortem examination confirmed sPAP and MDS.

**Lessons:**

In patients with CTD-ILD receiving immunosuppressive therapy, marked and progressive elevation of KL-6 should prompt reconsideration of the diagnosis, even when bronchoscopy does not demonstrate PAP and anti–GM-CSF antibodies are negative. Early recognition is essential to avoid inappropriate intensification of immunosuppression and further impairment of alveolar macrophage function.

## 1. Introduction

Pulmonary alveolar proteinosis (PAP) is a rare diffuse lung disease characterized by intra-alveolar accumulation of surfactant due to impaired clearance by alveolar macrophages.^[1]^ PAP is classified into congenital, autoimmune, and secondary forms. According to recent European Respiratory Society guidelines, the diagnosis of PAP is based on characteristic computed tomography findings and bronchoalveolar lavage cytology or lung histology, while identification of specific etiologies requires anti–granulocyte-macrophage colony-stimulating factor (GM-CSF) antibody testing or genetic analysis.^[2]^ Secondary PAP (sPAP) occurs in association with underlying conditions such as hematologic disorders, infections, and prolonged immunosuppressive therapy and is thought to result from quantitative or functional impairment of alveolar macrophages.^[1–3]^ Unlike autoimmune PAP (aPAP), sPAP lacks specific serological markers and is often associated with a poorer prognosis, making early diagnosis particularly challenging.

Adult-onset Still’s disease (AOSD) is a systemic autoinflammatory disorder that may be complicated by interstitial lung disease (ILD) and often requires prolonged treatment with glucocorticoids, biologic disease-modifying antirheumatic drugs, and Janus kinase (JAK) inhibitors.^[4–6]^ In patients with connective tissue disease–associated ILD (CTD-ILD), worsening respiratory findings are commonly interpreted as progression of the underlying ILD, leading to escalation of immunosuppressive therapy. However, serum Krebs von den Lungen-6 (KL-6), widely used as a biomarker of ILD activity, may also show marked elevation in PAP and reflect alveolar epithelial injury rather than inflammatory progression.^[7]^

Distinguishing progression of CTD-ILD from the development of PAP is particularly difficult when disease-specific activity markers are absent and anti–granulocyte-macrophage colony-stimulating factor (GM-CSF) antibodies are negative.

Here, we report an autopsy-confirmed case of sPAP developing during treatment for AOSD-associated ILD. In previously reported cases of PAP developing during treatment for autoimmune disease, newly developed pulmonary abnormalities or characteristic BAL and serological findings facilitated diagnostic recognition.^[8–12]^ In contrast, the present case was particularly challenging because preexisting ILD and repeated bronchoscopic findings of OP supported the interpretation of ILD progression, while anti–GM-CSF antibody testing was negative.

## 2. Case presentation

The patient was a 68-year-old Japanese woman with a smoking history of approximately 30 pack-years. At disease onset in 2022, she developed fever and rash after taking ampicillin. Skin biopsy findings suggested either adult-onset Still’s disease (AOSD) or pustular psoriasis. She subsequently developed OP, which improved with prednisolone in combination with cyclosporine A, and was referred to our hospital for evaluation of suspected CTD-ILD. On admission, laboratory findings showed leukocytosis, anemia, thrombocytosis, elevated C-reactive protein levels, and a serum KL-6 level of 190 IU per liter. Chest computed tomography (CT) revealed consolidations and ground-glass opacities predominantly in the lower lobes (Fig. 1A). Bronchoscopy performed in January 2022 showed nonmilky BAL fluid obtained from the lingular segment. TBLB specimens obtained twice from the left B8bi showed intra-alveolar fibrin deposition with mild alveolar wall thickening, consistent with OP, without findings suggestive of PAP. During follow-up, the patient developed persistent fever, rash, polyarthritis, hyperferritinemia, and elevated CRP levels. Although she fulfilled the 2010 ACR/EULAR classification criteria for rheumatoid arthritis (RA),^[13]^ a second skin biopsy demonstrated parakeratosis and superficial neutrophilic microabscesses consistent with AOSD, and she fulfilled the Yamaguchi criteria.^[14]^ Therefore, AOSD-associated ILD was considered more likely than RA-associated ILD. Tocilizumab was initiated but proved ineffective, and treatment was switched to upadacitinib. Clinical remission was achieved and glucocorticoids (GCs) were discontinued.

**Figure 1.**
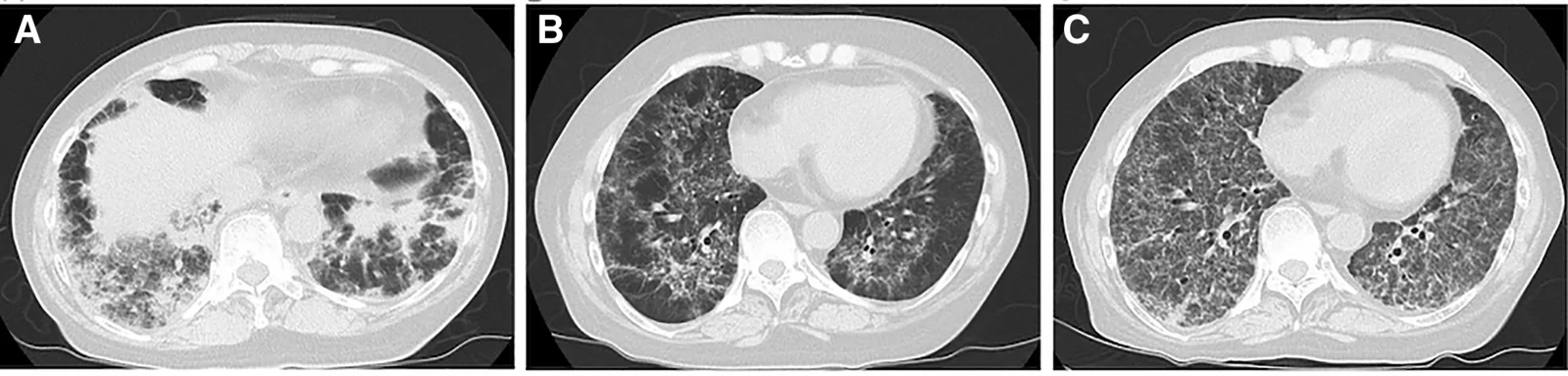
Serial chest computed tomography (CT) findings. (A) At initial presentation in 2022, reticular opacities with patchy consolidation are seen in both lungs. (B) During disease relapse in 2023, CT shows a subpleural-sparing distribution with ground-glass opacities and traction bronchiectasis, without radiological features typical of PAP. (C) One month before death in 2024, diffuse ground-glass opacities with interlobular septal thickening, forming a “crazy-paving” pattern, are observed bilaterally without marked reduction in lung volume.

In the following year, the disease relapsed, and GCs and methotrexate were reintroduced. Although the initial consolidations resolved, new diffuse ground-glass opacities with an airway-centered distribution appeared (Fig. 1B). Serum KL-6, which had been 190 IU per liter at initial presentation, increased to 1867 IU per liter. Because these findings raised concern for progression of ILD, bronchoscopy was repeated to reassess the diagnosis. The second bronchoscopy, performed in July 2023, again yielded nonmilky BAL fluid. TBLB specimens were obtained twice from the right B8a and once from the right B3a3 and showed intra-alveolar organization without evidence of PAP or infection. Laboratory testing revealed leukocytosis, mild anemia, and thrombocytosis. Hematologic abnormalities had been present since the initial evaluation but were initially considered to reflect systemic inflammation. A peripheral blood smear subsequently showed dysplastic changes, prompting bone marrow examination in August 2023, which demonstrated myelodysplastic syndrome (MDS)-like dysplasia. However, a definitive diagnosis of MDS was not established during life. After exclusion of opportunistic infections, immunosuppressive therapy was continued for presumed CTD-ILD. Tacrolimus, intravenous immunoglobulin, rituximab, and intravenous cyclophosphamide were administered sequentially. The clinical course was characterized by repeated exacerbations and partial remissions.

From the autumn of the following year, oxygen demand progressively increased, and serum KL-6 levels rose markedly to 19,333 IU per liter, without parallel elevation of CRP or ferritin. Chest CT revealed diffuse ground-glass opacities with interlobular septal thickening, forming a crazy-paving pattern throughout both lungs (Fig. 1C). Respiratory symptoms were limited to a mild cough. Repeat bronchoscopy was not feasible because of worsening respiratory failure requiring high-flow oxygen therapy with a fraction of inspired oxygen (FiO_2_) of 60%. Anti–GM-CSF antibody testing was performed because PAP was considered, but the result was negative. The major diagnostic and therapeutic events are summarized in Table 1.

**Table 1:** Summary of the clinical course and diagnostic evaluation.

Date	KL-6 (IU/L)	Radiological findings	BAL/TBLB findings	Hematologic evaluation	Treatment/ major event
Jan 2022	190	Consolidation and GGO	Nonmilky BAL fluid from the lingular segment; 2 TBLB specimens from left B8bi showing findings consistent with OP; no findings suggestive of PAP	Hematologic abnormalities considered related to systemic inflammation	PSL + CyA
Jul 2023	1867	New airway-centered GGO	Nonmilky BAL fluid from right B5a; 3 TBLB specimens (2 from right B8a and 1 from right B3a3) showing OP/organization without evidence of PAP or infection	–	Immunosuppressive therapy continued for presumed CTD-ILD
Aug 2023	–	–	–	Bone marrow examination showing MDS-like dysplasia; definitive MDS not established	Further immunosuppressive therapy
Apr 2024	19,333	Diffuse GGO/ crazy paving	Repeat bronchoscopy not feasible because of respiratory failure	–	Anti–GM-CSF antibody negative; progressive respiratory failure
Autopsy	–	–	sPAP confirmed histopathologically	MDS confirmed	Death

BAL = bronchoalveolar lavage; CTD-ILD = connective tissue disease–associated interstitial lung disease; CyA = cyclosporine A; GGO = ground-glass opacity; GM-CSF = granulocyte-macrophage colony-stimulating factor; KL-6 = Krebs von den Lungen-6; MDS = myelodysplastic syndrome; OP = organizing pneumonia; PAP = pulmonary alveolar proteinosis; PSL = prednisolone; sPAP = secondary pulmonary alveolar proteinosis; TBLB = transbronchial lung biopsy.

In early 2024, the patient’s respiratory condition rapidly deteriorated, and she died of respiratory failure (Fig. 2). After obtaining consent from her family, an autopsy was performed. Histopathological examination revealed alveolar spaces filled with periodic acid–Schiff–positive foamy macrophages and cholesterol clefts, confirming sPAP (Fig. 3). Bone marrow histology demonstrated increased blasts and dysplastic megakaryocytes, consistent with MDS (Fig. 4).

**Figure 2.**
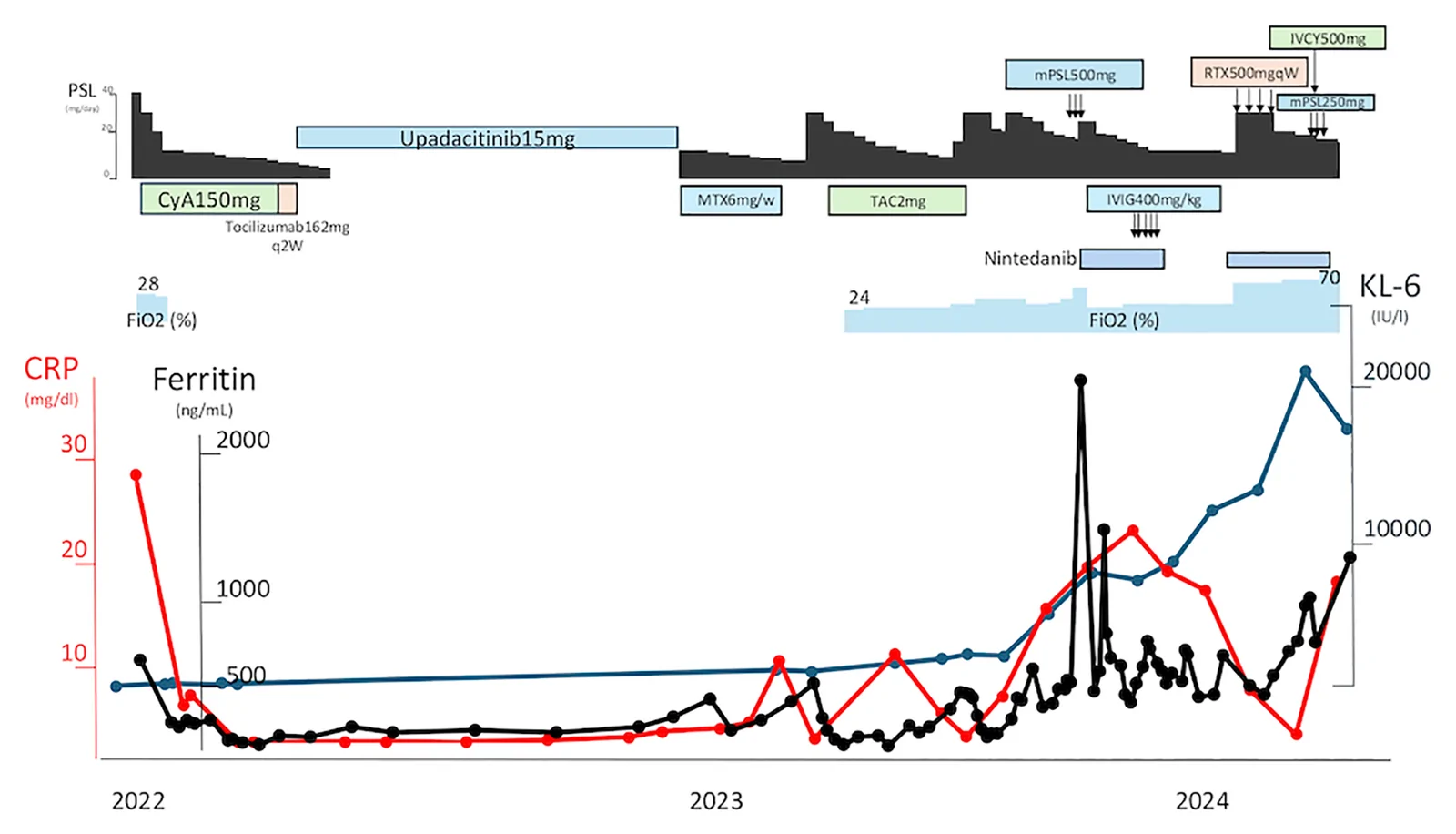
Clinical course from the onset of interstitial lung disease associated with AOSD to death. The top panel shows the administered medications, followed by the changes in FiO_2_. The line graph depicts the trends of CRP (red), ferritin (black), and KL-6 (blue) levels.CRP = C-reactive protein, CyA = cyclosporine A, FiO_2_ = fraction of inspired oxygen, IVCY = intravenous cyclophosphamide, IVIG = intravenous immunoglobulin, KL-6 = Krebs von den Lungen-6., mPSL = methylprednisolone, MTX = methotrexate, PSL = prednisolone, RTX = rituximab, TAC = tacrolimus.

**Figure 3.**
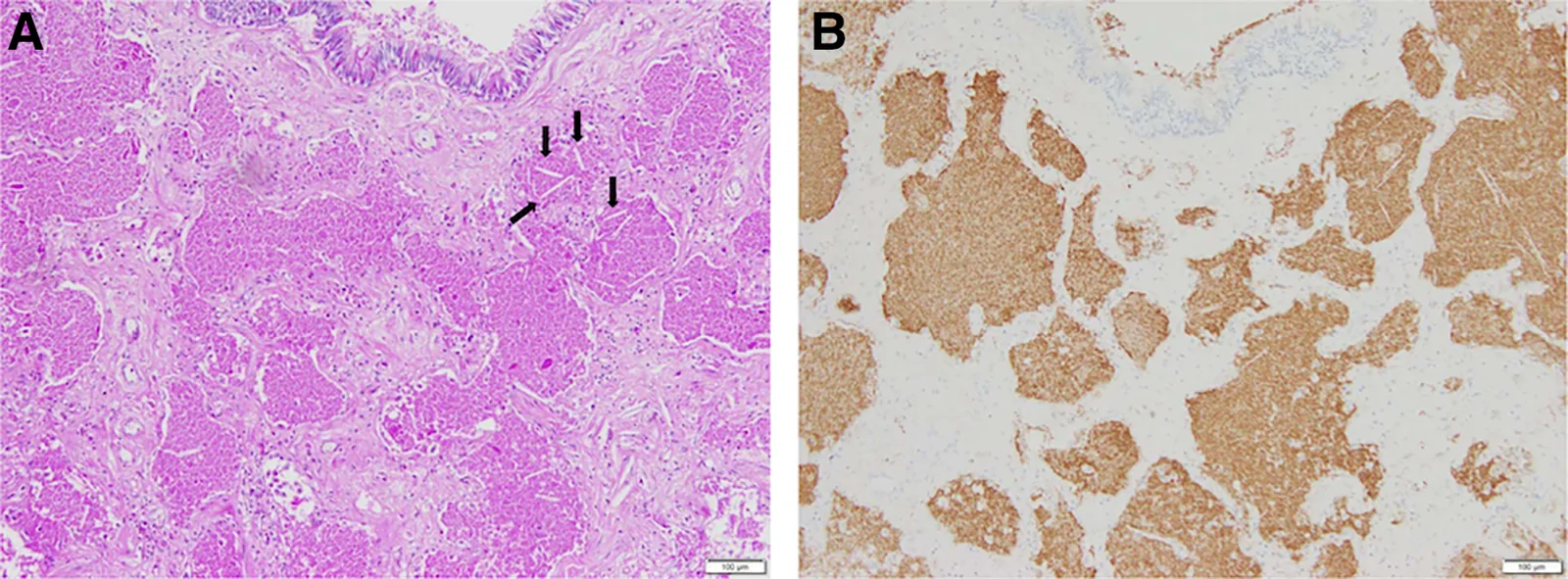
Histopathological findings of the lung at autopsy, leading to the diagnosis of pulmonary alveolar proteinosis (PAP). (A) The alveolar spaces are filled with fine granular material that is positive with periodic acid–Schiff (PAS) staining, admixed with cholesterol clefts (arrows) (PAS stain). (B) The fine granular material shows positive immunoreactivity for surfactant protein A (SP-A).

**Figure 4.**
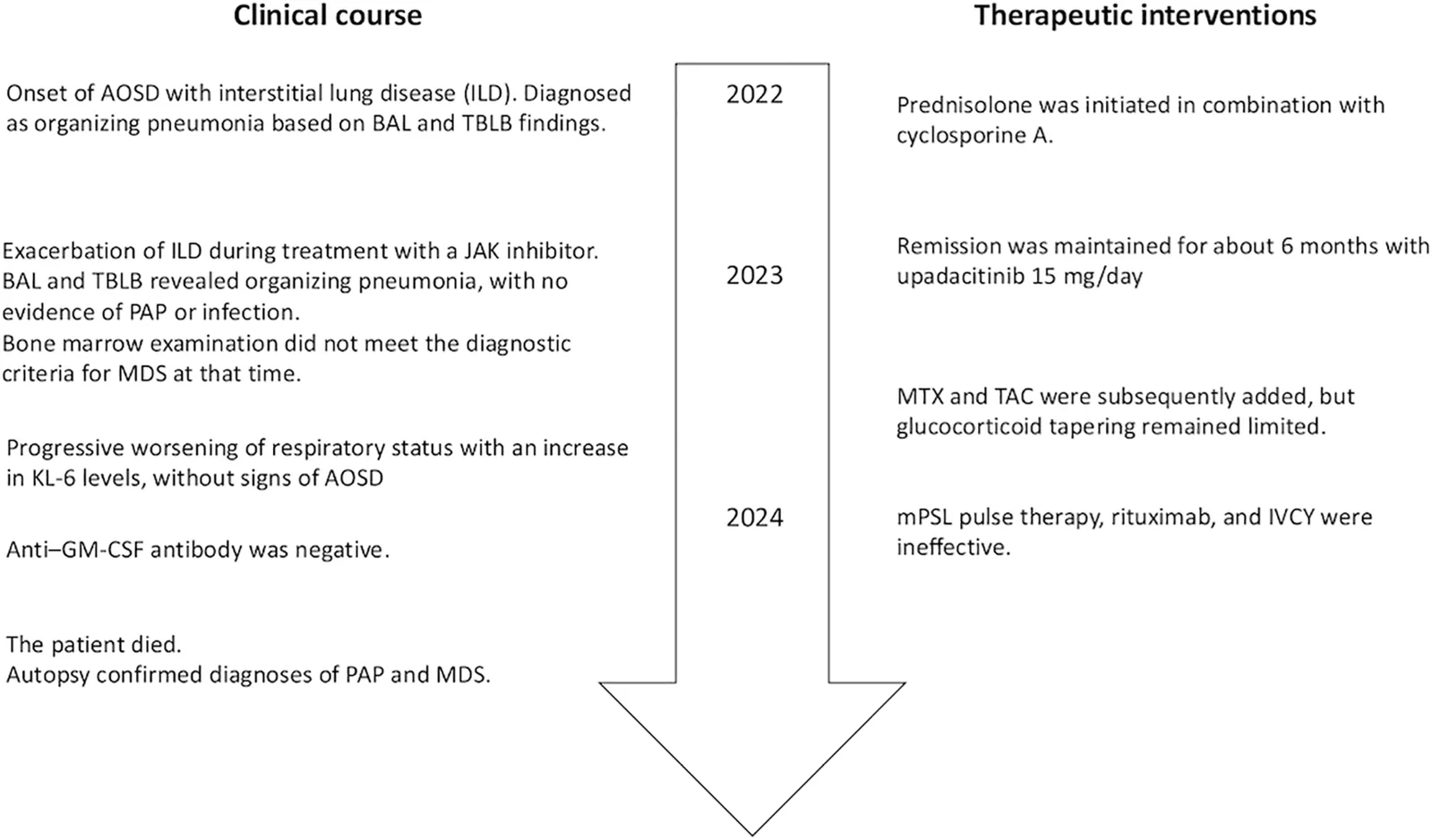
Timeline of the case. AOSD = adult-onset Still’s disease, BAL = bronchoalveolar lavage, GM-CSF = granulocyte–macrophage colony-stimulating factor, IVCY = intravenous cyclophosphamide, KL-6 = Krebs von den Lungen-6, MDS = myelodysplastic syndrome, mPSL = methylprednisolone, MTX = methotrexate, PAP = pulmonary alveolar proteinosis, TAC = tacrolimus, TBLB = transbronchial lung biopsy.

## 3. Discussion

This case highlights a diagnostic challenge in the management of CTD-ILD. In patients with worsening respiratory findings during treatment for CTD-ILD, marked elevation of KL-6 may not necessarily indicate progression of ILD alone. When KL-6 levels rise disproportionately and radiologic findings change despite previous bronchoscopic examinations being nondiagnostic for PAP, the diagnosis should be reconsidered even if anti–GM-CSF antibodies are negative.

At disease onset, lung pathology was consistent with OP. During the clinical course, the radiologic pattern changed from consolidation to diffuse ground-glass opacities with an airway-centered distribution and later to a crazy-paving pattern. Previous comparative studies have shown that diffuse ground-glass opacities are more typical of sPAP, whereas the classic crazy-paving pattern is more frequently observed in aPAP.^[15]^ A retrospective study also reported that CT findings in sPAP may predominantly consist of ground-glass opacities without prominent interlobular septal thickening.^[16]^ In the present case, the radiologic findings were not entirely characteristic of either pattern, which may have further complicated early recognition. Repeat bronchoscopy demonstrated OP without evidence of PAP, supporting the interpretation of progressive CTD-ILD. In the terminal stage, imaging became more suggestive of an alveolar filling process, but bronchoscopy was no longer feasible.

In aPAP, detection of anti–GM-CSF antibodies facilitates diagnosis. In contrast, sPAP lacks specific serological markers, making recognition more challenging.^[2]^ Several reports have described aPAP emerging during treatment for ILD, in which positive anti–GM-CSF antibodies and characteristic BAL findings enabled definitive diagnosis.^[8–12]^ Secondary PAP, however, may occur despite negative antibody testing.^[16–18]^ In the present case, repeated bronchoscopy demonstrated only OP without characteristic features of PAP. A recent case report described sPAP complicating AOSD, in which new pulmonary infiltrates developed during immunosuppressive therapy and were confirmed by biopsy in the absence of anti–GM-CSF antibodies.^[17]^ Unlike the present case, pulmonary involvement was not preexisting interstitial lung disease, which may have facilitated diagnostic reconsideration. This distinction underscores how preexisting CTD-ILD may obscure recognition of PAP when disease-specific markers are lacking.

The TBLB specimens obtained in the present case were considered adequate for histopathological evaluation. However, the exact timing and spatial distribution of sPAP before the terminal stage remain uncertain. Although neither BAL nor TBLB demonstrated characteristic features of PAP, focal or patchy involvement and consequent sampling error cannot be excluded. The ERS guidelines note that histological examination may be nondiagnostic because of patchy involvement and that lung biopsy is not routinely recommended in the diagnostic workup of suspected PAP.^[2]^ Therefore, the negative bronchoscopic findings in the present case cannot establish whether PAP was absent at the time of either examination.

KL-6 is associated with disease activity and poor prognosis in PAP.^[7]^ In this case, KL-6 increased from 1867 to 19,333 IU per liter, levels uncommon in typical CTD-ILD. Similar cases have shown that persistent KL-6 elevation during immunosuppressive therapy may indicate underlying sPAP.^[19]^ These observations emphasize the importance of recognizing disproportionate or progressive KL-6 elevation when clinical and radiologic findings appear discordant.

Autopsy revealed MDS, which is known to be associated with sPAP and adverse outcomes.^[20]^ Hematologic malignancies have been reported to predispose to sPAP through impaired macrophage differentiation and function.^[21]^ Experimental studies have shown that JAK inhibition suppresses GM-CSF–mediated signaling and impairs innate immune cell function.^[22]^ An autopsy case of sPAP associated with primary myelofibrosis treated with the JAK1/2 inhibitor ruxolitinib has been reported, suggesting a potential link between JAK inhibition and impaired surfactant clearance.^[23]^ Although direct causality cannot be established, cumulative immunomodulatory therapy may contribute to impaired alveolar macrophage function. A retrospective cohort study of patients with aPAP demonstrated that corticosteroid therapy was associated with significant worsening of disease severity, particularly at higher doses.^[24]^ In the present case, GCs were minimized whenever clinically feasible. However, prolonged exposure to multiple immunosuppressive agents, including JAK inhibition, may still have compromised alveolar macrophage homeostasis and contributed to the development of sPAP.

When CTD-ILD worsens during immunosuppressive therapy, particularly in the presence of marked or progressive elevation of KL-6, clinicians should reconsider the diagnosis rather than automatically escalating treatment. If sPAP remains clinically suspected despite a previous nondiagnostic bronchoscopy and negative anti–GM-CSF antibody testing, closer radiological follow-up and repeat BAL should be considered. Serial CT assessment may help identify evolving radiological features suggestive of PAP and prompt further diagnostic evaluation, consistent with the important role of CT in the diagnosis and assessment of disease progression in PAP.^[2]^ Periodic acid–Schiff (PAS) staining of BAL fluid is an important component of the diagnostic evaluation for suspected PAP.^[2]^ This is particularly relevant to the present case because PAS staining was not performed on BAL fluid during either bronchoscopic examination. Lung biopsy should not be performed routinely; however, if BAL and noninvasive investigations remain nondiagnostic despite persistent clinical suspicion, tissue sampling may be considered after assessment of the expected diagnostic benefit and procedural risk.^[2]^ Transbronchial lung cryobiopsy can provide larger specimens than conventional TBLB and has been reported to facilitate the diagnosis of PAP, although evidence remains limited.^[25,26]^ Surgical lung biopsy should be reserved for carefully selected cases when less invasive approaches remain nondiagnostic.^[2]^ In the present case, respiratory deterioration occurred during the second conventional TBLB procedure. This experience underscores the importance of carefully assessing respiratory status and procedural risk when considering further invasive diagnostic procedures, including cryobiopsy.

## 4. Conclusion

Secondary pulmonary alveolar proteinosis can emerge during treatment for CTD-ILD and may be overlooked when worsening findings are interpreted as progression of ILD. Because sPAP is associated with poor prognosis and may follow a rapidly progressive course,^[3]^ timely recognition is essential. Even when previous bronchoscopic findings are nondiagnostic and anti–GM-CSF antibodies are negative, disproportionate elevation of KL-6 should prompt diagnostic reassessment. Early recognition is crucial to avoid unnecessary intensification of immunosuppressive therapy and further impairment of alveolar macrophage function.

## Author contributions

**Conceptualization:** Takashi Yamane.

**Investigation:** Takashi Yamane, Taisuke Fujita, Atsushi Onoe, Chihiro Ichikawa.

**Writing – original draft:** Takashi Yamane.

**Writing—review & editing:** Takashi Yamane, Taisuke Fujita, Atsushi Onoe, Chihiro Ichikawa.
